# Early Results of an Initiative to Assess Exposure to Firearm Violence in Ambulatory Care: Descriptive Analysis of Electronic Health Record Data

**DOI:** 10.2196/47444

**Published:** 2024-02-05

**Authors:** Nicole Cook, Megan Hoopes, Frances M Biel, Natalie Cartwright, Michelle Gordon, Marion Sills

**Affiliations:** 1 OCHIN Portland, OR United States; 2 Department of Mathematics Norwich University Northfield, VT United States

**Keywords:** gun violence, firearm injury, surveillance, primary care, public health, ambulatory care, electronic health record, violence, burden, emergency department, data, risk factor

## Abstract

**Background:**

Current research on firearm violence is largely limited to patients who received care in emergency departments or inpatient acute care settings or who died. This is because standardized disease classification codes for firearm injury only represent bodily trauma. As a result, research on pathways and health impacts of firearm violence is largely limited to people who experienced acute bodily trauma and does not include the estimated millions of individuals who were exposed to firearm violence but did not sustain acute injury. Assessing and collecting data on exposure to firearm violence in ambulatory care settings can expand research and more fully frame the public health issue.

**Objective:**

The aim of the study is to evaluate the demographic and clinical characteristics of patients who self-reported exposure to firearm violence during a behavioral health visit.

**Methods:**

This study assessed early data from an initiative implemented in 2022 across a national network of ambulatory behavioral health centers to support trauma-informed care by integrating structured data fields on trauma exposure into an electronic health record behavioral health patient assessment form (SmartForm), as such variables are generally not included in standard outpatient medical records. We calculated descriptive statistics on clinic characteristics, patient demographics, and select clinical conditions among clinics that chose to implement the SmartForm and among patients who reported an exposure to firearm violence. Data on patient counts are limited to positive reports of exposure to firearm violence, and the representativeness of firearm exposure among all patients could not be calculated due to unknown variability in the implementation of the SmartForm.

**Results:**

There were 323 of 629 (51%) clinics that implemented the SmartForm and reported at least 1 patient exposed to firearm violence. In the first 11 months of implementation, 3165 patients reported a recent or past exposure to firearm violence across the 323 clinics. Among patients reporting exposure, 52.7% (n=1669) were male, 38.8% (n=1229) were Black, 45.7% (n=1445) had posttraumatic stress disorder, 37.5% (n=1186) had a substance abuse disorder (other than nicotine), and 11.7% (n=371) had hypertension.

**Conclusions:**

Current research on firearm violence using standardized data is limited to acute care settings and death data. Early results from an initiative across a large network of behavioral health clinics demonstrate that a high number of clinics chose to implement the SmartForm, resulting in thousands of patients reporting exposure to firearm violence. This study demonstrates that collecting standardized data on firearm violence exposure in ambulatory care settings is feasible. This study further demonstrates that resultant data from ambulatory settings can be used for meaningful analysis in describing populations affected by firearm violence. The results of this study hold promise for further collection of structured data on exposure to firearm violence in ambulatory settings.

## Introduction

The escalation in firearm injury in the United States is reflected in epidemiologic trends, including the emergence of firearm violence as the leading cause of mortality in children ages 1-18 years since 2020—yet the public health burden of firearm injury is severely undercounted [1-3]. Current incidence and prevalence of firearm injury are largely limited to acute injury data from hospitals and emergency departments and death data. This is because clinical classification codes (International Classification of Diseases) for firearm injury currently only represent acute bodily trauma [4-9]. While millions of children and adults are estimated to have experienced primary or secondary exposure to firearm violence (direct witness of firearm violence or the acute aftermath), there is no standardized data collection system to support surveillance of this public health problem [10-12]. As a result, pathways, risk factors, and intervention strategies following exposure to firearm violence are poorly understood [13]. Collecting broader data on exposure to firearm violence and the physical and psychological injury following exposure is critically needed to identify risk factors and disease pathways, frame the full impact of firearm violence on society, and inform the development of comprehensive treatments for people impacted by firearm violence [12]. Ambulatory electronic health record (EHR) data are potentially rich sources of injury and exposure data that can broaden our understanding of the spectrum of health impacts of firearm violence.

In 2022, a large national network of community-based behavioral health safety–net clinics serving racial and ethnic minority groups, low-income and underserved rural communities, sexual and gender minorities, and other health disparate populations implemented a customized data collection tool (SmartForm) in their shared EHR to collect trauma history as part of the behavioral health assessment process for clients. All network clinics are located in medically underserved areas and serve minoritized populations most likely to experience disparate community firearm violence [14,15]. The SmartForm implementation included an associated workflow to collect standardized data on recent or past exposure to firearm violence among patients presenting for behavioral health care [16]. Implementation of the trauma SmartForm is part of a larger quality improvement initiative to optimize EHR solutions to support trauma-informed care across the network [17]. To our knowledge, this initiative is the first large-scale initiative to collect standardized firearm violence exposure data in outpatient care settings. In this paper, we present early results on exposure to firearm violence from the first 11 months of implementation of the SmartForm.

## Methods

### Study Design

We used EHR data from the OCHIN multistate network of community-based primary and behavioral health care clinics [16]. OCHIN is a nonprofit health care innovation center that offers a fully hosted, highly customized instance of Epic practice management and EHR solutions to 140 members representing 1071 clinic sites, 629 (59%) of whom provide behavioral health services. All OCHIN members are trained on common workflows. Data on “exposure to gun violence” are incorporated as an optional standardized, reportable field as part of the behavioral health trauma SmartForm within the patient history section of the EHR used by behavioral health clinicians. While associated workflows are recommended, clinics and clinicians within the network have full autonomy to determine their use (or nonuse) of the SmartForm. As a quality improvement initiative, it is accepted that the use of the SmartForm is not consistent across all network clinics.

EHR reportable data include “recent exposure to gun violence” and “past exposure to gun violence.” Our data set represents cross-sectional data from February 2022, when the trauma SmartForm was implemented in the EHR, to December 2022. The SmartForm in use during this time only collected patient-reported “yes” responses to firearm violence exposure; there was no ability to document “no” responses. As a result, there is no denominator of total patients assessed for exposure and no practical method for estimating the prevalence of self-reported firearm violence exposure within the population.

We identified all active patients with documentation of a recent or past exposure to firearm violence and extracted data on demographics (age, sex, etc) and the prevalence of a select, predefined list of active health problems including behavioral health diagnoses, diabetes, and hypertension [18]. The list of active health problems was extracted from the patient problem list within the EHR.

### Ethical Considerations

This project used a deidentified data set from a data repository of clinical and administrative data of all patients seen in the OCHIN network. The data repository is under institutional review board oversight with Advarra (Pro00060082) and includes a waiver of consent and authorization. This project was reviewed by Norwich University Institutional Review Board (00005859) and OCHIN and met exemption criteria under 45 CFR 46.

## Results

During the study period, there were 129 active OCHIN member organizations with 629 behavioral health clinic sites. Among those, 91 (70.5%) member organizations and 323 (51.3%) clinics implemented the SmartForm with at least 1 patient reporting exposure to firearm violence per clinic. Between February 1 and December 31, 2022, these clinics documented between 19 and 33,067 (median 1066, IQR 356-4309) patients with firearm violence exposure, with a median age of 17 (IQR 7-64) years. Of these, a median of 43% (IQR 39%-49%; range 2%-92%) of patients were male. Across the 323 clinics, 3165 behavioral health patients had patient-reported and clinician-documented recent or past exposure to firearm violence noted in their EHR between February 2022 and December 2022 (Table 1). Among patients with noted firearm exposure, 52.7% (n=1669) were male, 41.4% (n=1308) were between 12 and 34 years of age, 11.5% (n=364) self-reported having a sexual orientation other than straight, 38.3% (n=1229) were Black or African American, and 42.5% (n=1334) were uninsured. The median age of patients with noted exposure to firearm violence was 20 (IQR 7-64; range 3-89) years. When described by behavioral health diagnosis, 45.7% (n=1445) had a diagnosis of posttraumatic stress disorder, and 37.5% (n=1886) had a substance abuse disorder (excluding nicotine dependence). A large percentage of patients with a documented exposure had hypertension (n=371, 11.7%), and 66.7% (n=2110) had 7 or more clinic visits (for either behavioral health or primary care) during 2022. Each clinic determines which social risks, if any, they measure. Among patients screened for social determinants of health, 69.8% (n=1213) reported one or more social risks such as food insecurity or transportation insecurity.

**Table 1:** Demographic and active conditions of patients with self-reported exposure to recent or past firearm violence across 323 ambulatory behavioral health clinics (February to December 2022; N=3165).

		Patients reporting exposure, n (%)
**Age group (years)**		
	0-11	56 (1.8)
	12-17	193 (6.1)
	18-24	334 (10.6)
	25-34	781 (24.7)
	35-49	1027 (32.4)
	50+	774 (24.5)
**Assigned sex^a^**		
	Female	1496 (47.3)
	Male	1669 (52.7)
**Gender identity**		
	Man	1433 (45.3)
	Woman	1295 (40.9)
	Transman and transwoman	43 (1.4)
	Genderqueer; reported other gender identity	51 (1.6)
	Decline or unknown	343 (10.8)
**Sexual orientation**		
	Straight	2226 (70.3)
	Gay, lesbian, bisexual, multiple; reported other sexual orientation	364 (11.5)
	Decline or unknown	575 (18.2)
**Race**		
	American Indian or Alaska Native	77 (2.4)
	Asian	49 (1.5)
	Black or African American	1229 (38.8)
	Native Hawaiian or other Pacific Islander	18 (0.6)
	White	1368 (43.2)
	Multiple races	51 (1.6)
	Decline or unknown	373 (11.8)
**Ethnicity**		
	Hispanic or Latinx	543 (17.2)
	Non-Hispanic or Latinx	2388 (75.5)
	Unknown	234 (7.4)
**Preferred language**		
	English	2958 (93.5)
	Spanish	146 (4.6)
	Other or unknown	61 (1.9)
**Payor type**		
	Medicaid	1406 (44.4)
	Uninsured or other public insurance	1344 (42.5)
	Private	225 (7.1)
	Medicare	190 (6)
Veteran status (among adults age 18 years and older)		63 (2.2)
Homeless		131 (4.1)
**Ever screened for social determinants of health^b^**		1738 (54.9)
	Reported one or more adverse social determinants of health (those ever screened)	1213 (69.8)
Assigned primary care provider		2047 (64.7)
**Number of clinic visits in 2022**		
	0-2	247 (7.8)
	3-6	808 (25.5)
	7+	2110 (66.6)
**Active behavioral health conditions**		
	PTSD^c^ and reaction to severe stress (F43)	1445 (45.7)
	Other anxiety disorders (F41)	1268 (40.1)
	Substance use disorders (excluding nicotine dependence; F10-F16, F18, and F19)	1186 (37.5)
	Major depressive disorder (F33)	753 (23.8)
	Depressive episode (F32)	683 (21.6)
**Active medical conditions**		
	Hypertension (I10)	371 (11.7)
	Lipid disorders (E78)	316 (10)
	Type 2 diabetes (E11)	164 (5.2)

^a^A small number of patients with other or unknown sex were proportionally distributed to the female and male categories to avoid reporting small cell counts.

^b^Social determinants of health is a broad category, and each clinic has the autonomy to determine which, and how many, social determinants of health they collect for patients. Adverse social determinants of health include social risks including, but not limited to, food insecurity, housing insecurity, and transportation insecurity.

^c^PTSD: posttraumatic stress disorder.

## Discussion

### Principal Findings

Current firearm injury research, largely limited to acute injury and death, has many shortcomings [19] that can potentially be overcome by expanding the collection of standardized data on firearm violence exposure in ambulatory care settings. A review of EHR data from safety-net clinics within the OCHIN network, which initiated a standardized question on firearm violence exposure as part of collecting information on patient history during behavioral health care visits, demonstrates that data on firearm violence can be collected as part of routine behavioral health care. EHR data include patient-level variables not currently available in common firearm injury data sets (Healthcare Cost & Utilization Project; National Vital Statistics System/Web-Based Injury Statistics Query and Reporting System) used for research, including gender identity, sexual orientation, social determinants of health, experiencing homelessness, health care use, behavioral health, and chronic disease comorbidities [20-22].

### Strengths and Limitations

Expanding the collection of firearm violence exposure data in outpatient EHRs opens new opportunities in firearm violence research; these novel data may be leveraged to support more precise prevalence estimates of firearm violence exposure and injury, assess population-level associations between exposure and medical or behavioral health outcomes, and support the application of machine learning to develop predictive analytics for treatment planning [1,12,23].

Some limitations of this analysis arise from the nonmandatory nature of the data collection form. As OCHIN implemented the EHR trauma SmartForm as part of a quality improvement behavioral health EHR optimization initiative, clinics and clinicians within the network had full autonomy to determine their use (or nonuse) of the SmartForm. As the SmartForm is not consistently used across all OCHIN member clinics, the data presented here are representative of neither OCHIN’s patient population nor clinician’s behavior. In addition, the SmartForm currently in use only collects patient-reported “yes” responses to firearm violence exposure; there is no place to document “no” responses. As a result, there is no denominator of total patients assessed for exposure and no practical method for estimating the prevalence of self-reported firearm violence exposure within the population.

This study broadens the discussion around firearm injury by demonstrating that standardized data on firearm violence exposure can be collected in outpatient settings. Such data can extend our knowledge of the burden of firearm violence, which is typically limited to acute care settings including inpatient and emergency department data or mortality data, by incorporating a broader understanding of both exposure to firearm violence and firearm injury through the availability of rich demographic, clinical, psychological, and social risk data collected in ambulatory care EHRs.

### Future Directions

Results from this study demonstrate the potential value of screening for firearm violence exposure and suggest how collection of this novel data may be leveraged to support firearm injury surveillance, understand physical and psychological firearm violence sequelae, and support treatment. Ongoing work should include identifying opportunities for more systematic screening of exposure to firearm violence across health care settings.

## Abbreviations

EHR: electronic health record

## Data Availability

The data sets generated and analyzed during this study are not publicly available due to source patient-level data from multiple health systems, which have restrictions regarding the availability and rerelease of data under cross-institution agreements. Data are available from the corresponding author on reasonable request and with permission of all relevant parties.
